# Effects of a powered ankle-foot orthosis on perturbed standing balance

**DOI:** 10.1186/s12984-018-0393-8

**Published:** 2018-06-18

**Authors:** Amber R. Emmens, Edwin H. F. van Asseldonk, Herman van der Kooij

**Affiliations:** Department of Biomechanical Engineering, University of Twente, Drienerlolaan 5, Enschede, 7522 NB the Netherlands

**Keywords:** Standing balance, Ankle-foot orthosis, Balance control, Exoskeleton

## Abstract

**Background:**

Lower extremity exoskeletons are mainly used to provide stepping support, while balancing is left to the user. Designing balance controllers is one of the biggest challenges in the development of exoskeletons. The goal of this study was to design and evaluate a balance controller for a powered ankle-foot orthosis and assess its effect on the standing balance of healthy subjects.

**Methods:**

We designed and implemented a balance controller based on the subject’s body sway. This controller was compared to a simple virtual-ankle stiffness and a zero impedance controller. Ten healthy subjects wearing a powered ankle-foot orthosis had to maintain standing balance without stepping while receiving anteroposterior pushes. Center of mass kinematics, ankle torques and muscle activity of the lower legs were analyzed to assess the balance performance of the user and exoskeleton.

**Results:**

The different controllers did not significantly affect the center of mass responses. However, the body sway based controller resulted in a decrease of 29% in the biological ankle torque compared to the zero impedance controller and a decrease of 32% compared to the virtual-ankle stiffness. Furthermore, the soleus muscle activity of the left and right leg decreased on average with 8%, while the tibialis anterior muscle activity increased with 47% compared to zero impedance.

**Conclusion:**

The body sway based controller generated human-like torque profiles, whereas the virtual-ankle stiffness did not. As a result, the powered ankle-foot orthosis with the body sway based controller was effective in assisting the healthy subjects in maintaining balance, although the improvements were not seen in the body sway response, but in the subjects’ decreased biological ankle torques to counteract the perturbations. This decrease was a combined effect of decreased soleus muscle activity and increased tibialis anterior muscle activity.

## Background

Exoskeletons have been successfully used to provide assistance during walking. For people with lower extremity impairments the focus is generally on rehabilitation [1–5] and gait restoration [6–8], while exoskeletons for healthy people aim to augment strength and endurance [9–13]. A particular goal of exoskeleton studies is to reduce the effort of walking. A key challenge is to overcome the negative effects of the added weight of the exoskeleton. It was shown that when using passive or powered ankle-foot orthoses, it is possible to reduce the muscle activity of ankle plantar flexors [14, 15].

While healthy users are able to walk in wearable exoskeletons without assistive devices, for people suffering from neurological disorders such as a spinal cord injury this is difficult, if not impossible, due to affected balance. Except for the wearable exoskeleton “REX” (REX Bionics Ltd, New Zealand) [16], commercially available devices are used in combination with crutches for maintaining balance [3, 7, 17]. This implies that the users are supporting themselves and the exoskeleton in staying upright, although it would be desirable if the exoskeleton supported the user’s balance. Still, only a few studies have addressed the topic of exoskeleton balance control during walking [6, 18] or standing [19]. These studies mainly focused on controller implementation and not on its effect on the user. In maintaining balance, the user and the exoskeleton should work in harmony and therefore it is essential to incorporate knowledge about human balance control in the controller.

Several models have been suggested to describe the dynamics of human standing balance control, based on full-state feedback [20–24] or center of mass (CoM) motion [25–27]. For full-state feedback, the human is modeled as a multi-link system and the state vector generally consists of the angles and velocities of all the joints. How much each state contributes to a certain joint torque can be acquired by fitting experimental data to a model simulation. Through forward simulations it was shown that full-state feedback models are able to predict ankle and hip torques well in a linearized double-inverted pendulum model [22]. For CoM feedback, the whole body CoM of a human is generally assumed to sway like a single inverted pendulum. This model has proven to be sufficient for describing the balance strategy known as the “ankle strategy,” which is dominant in quiet stance and other simple balancing tasks [28]. Furthermore, previous studies have shown that CoM kinematics can be used to reproduce muscle activity patterns during both ankle and hip postural responses [25, 26].

Our goal is to use an exoskeleton for balancing during standing and walking and to assess the effect of the exoskeleton on the user. As a first step we tested balance controllers on a powered Ankle-Foot Orthosis (pAFO) and investigated standing balance in the sagittal plane. Since task-space goals, such as controlling CoM excursion, play an important role in standing balance, the proposed balance controller was based on CoM kinematics. The assistive torque that the pAFO had to deliver was calculated from the CoM kinematics according to the single inverted pendulum model. We compared this control law to a simple stiffness applied around the ankle, which is equivalent to controlling a single inverted pendulum model in joint-space. Furthermore, ankle stiffness corresponded to the largest feedback gain of the full-state feedback law for the ankle torque [21]. As such, the stiffness controller had the potential to positively affect balance performance, without introducing task-space complexity. The goal of this research is twofold: 1. to assess the effects of the controlled pAFO on the balance performance of the user; and 2. to assess the effects of the controlled pAFO on human effort, based on ankle joint kinetics and muscle activity of the ankle plantar and dorsiflexors. We hypothesized that, with the application of an assistive torque that is similar to the ankle torque humans generate when being perturbed, balance performance would improve, while the human contribution to the balance response would decrease.

## Methods

In this study, the assistive effect of the pAFO was tested by means of experiments in which subjects had to maintain balance, without stepping, while receiving external perturbations. Ethical approval for the experiments was given by the Human Research Ethics Committee of Delft University of Technology, the Netherlands.

### Subjects

Ten healthy subjects (three female, mass 71 ± 6 kg, height 1.81 ± 0.07 m, mean ± SD) participated in the experiment after giving written informed consent. Based on the size and stiffness characteristics of the pAFO, participants were selected who were taller than 1.65 m, weighed less than 84 kg, and had an EU shoe size between 36 and 44.

### Experimental setup

**Fig. 1:**
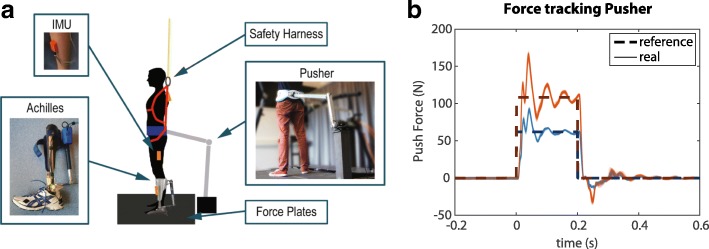
Experimental setup and perturbation profile. **a** Overview of the setup. In addition to the shown hardware, EMG sensors were placed on the lower legs. **b** The reference perturbation force profile of the Pusher and all generated perturbation forces (real) for a representative subject. There is little variation in the generated perturbation forces

Figure 1 shows a schematic overview of the experimental setup.

#### The Achilles Ankle-Foot Orthosis

**Fig. 2:**
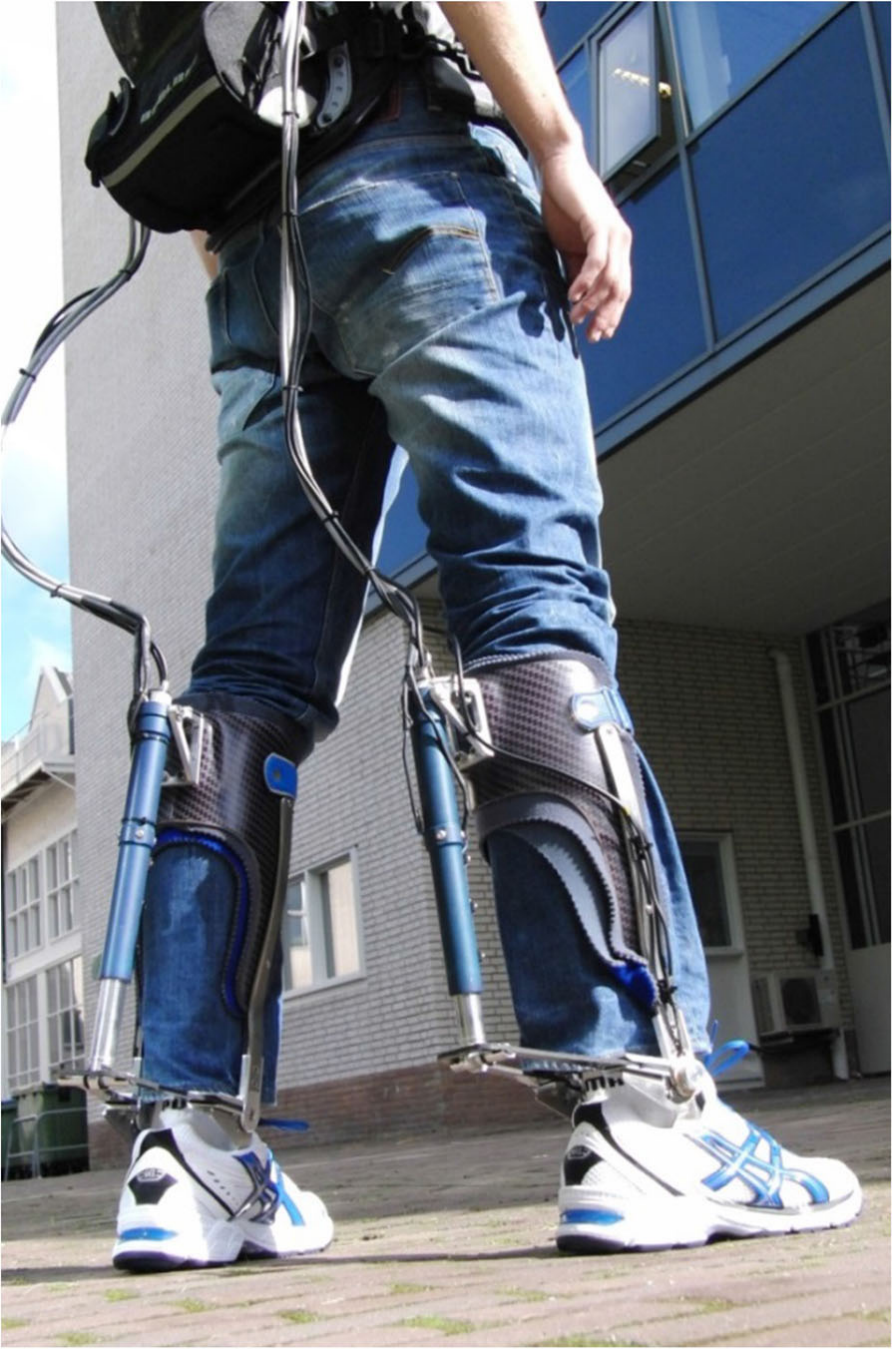
The Achilles ankle-foot orthosis

The Achilles [29] is a pAFO that can provide dorsi/plantar flexion torques to the ankle, while inversion and eversion are constrained. As shown in Fig. 2, the complete device consists of two orthoses (1.5 kg each) and a backpack. Each orthosis is actuated by a motor and ball-screw spindle. The spindle is connected to the rotational joint of the orthosis by a leaf spring, such that a translation of the spindle results in a spring deflection and a joint torque. To measure the motor stroke and the joint rotation, the device is equipped with an incremental encoder on the motor axis and an absolute encoder at the rotational joint. Since the spring stiffness is known (410 Nm/rad) and the spring deflection depends on the motor stroke and the joint rotation, the torque that is delivered by the Achilles can be estimated [29]. In the Zero Impedance (ZI) condition the spring deflection, and therefore the joint torque, is minimized. There is no compensation for the inertia of the device, but the inertia effects are small.

The Achilles backpack contains a PC (EtherCAT Master) that communicates with the orthoses and other hardware modules (EtherCAT slaves). EtherLab (IgH, Essen, Germany) is used to compile MATLAB/Simulink (2014b, The Mathworks, Natick, MA, USA) models into executables that can run on the real-time Linux EtherCAT master. During the experiments subjects did not wear the backpack, but it was hung behind them on parallel bars.

#### Perturbation device

An actuated perturbation device, referred to as “Pusher,” was used to provide forward perturbations at the pelvis. It consists of a motor (SMH60, Moog, Nieuw-Vennep, The Netherlands) located at the rear of the treadmill, connected to a horizontal push rod (0.8 m) through a lever arm (0.3 m), as shown on the right of Fig. 1a. At the end of the push rod a modified universal hip abduction brace (Distrac Wellcare, Hoegaarden, Belgium) was attached, which was tightly worn by the subjects. A load cell (model QLA131, FUTEK, Los Angeles, CA, USA) was mounted on the lever arm for torque sensing [30]. The rod was approximately horizontal, such that a motor torque would result in forward pushing or backward pulling of the subject. The motor was admittance controlled over Ethernet (User Datagram Protocol) at 1000 Hz, using xPC-target (The Mathworks, Natick, MA, USA).

#### Measurement equipment

To measure the orientation of the shank, thigh and trunk of the subject, three Xsens (Xsens Technologies B.V., Enschede, the Netherlands) MTx Inertial Measurement Units (IMUs) with integrated magnetometers were used. Each IMU was calibrated by defining its axis of rotation to correspond to the mediolateral axis of the subject. This calibration was done by letting each subject make a squat movement at the start of the first trial. The signals of the IMU’s were made available real-time on the EtherCAT Master through a RS232-EtherCAT converter box.

Besides motion data, ground reaction forces and moments were collected using a custom made split-belt instrumented treadmill (Y- Mill, Motek, Amsterdam, The Netherlands) that has force plates beneath each belt. Furthermore, the Delsys Trigno Lab Wireless EMG System (Delsys Inc., Natick, USA) was used to record EMG signals on the gastrocnemius medialis (GM), soleus (Sol) and tibialis anterior (TA). The EMG sensors were equipped with a fourth order Butterworth filter that rejected frequencies above 450 Hz.

### Control strategies

#### Body sway controller

The body sway controller, PD _com_, was a proportional-derivative controller that uses the CoM motion as an input. The control law for the PD _com_ is as follows:

1 $$\begin{array}{@{}rcl@{}}  \tau_{ach} = K_{P}\left(\theta_{{sway}_{d}}-\theta_{sway}\right)+K_{D}\left(\dot{\theta}_{{sway}_{d}}-\dot{\theta}_{sway}\right) \end{array} $$

where *τ*_*ach*_ is the desired support torque that the Achilles should deliver, *K*_*P*_ and *K*_*D*_ are proportional and derivative gains respectively, *θ*_*sway*_ is the body sway angle that is dependent on the CoM location, and subscript *d* indicates a desired value. A complete list of symbols is provided in Table 1.

**Table 1:** List of symbols

Symbol	Meaning
*F*_*Cx*_	Sum of the horizontal ground reaction forces
*F*_*Cy*_	Sum of the vertical ground reaction forces
*g*	Gravitational acceleration
*h*_*A*_	Ankle height w.r.t. the ground
*K*	Proportional gain of the virtual-ankle stiffness controller
*K*_*D*_	Derivative gain of the body sway controller
*K*_*P*_	Proportional gain of the body sway controller
*l*_*com*_	Height of the center of mass in upright stance w.r.t. the ankle
*M*	Subject mass
*p*_*x*_	Center of pressure in anteroposterior direction w.r.t. the ankle
*θ*_*sway*_	Body sway angle
*τ*_*ach*_	Desired support torque
*τ*_*tot*_	Total torque applied to the ankle
*ϕ*_*A*_	Ankle joint angle

**Fig. 3:**
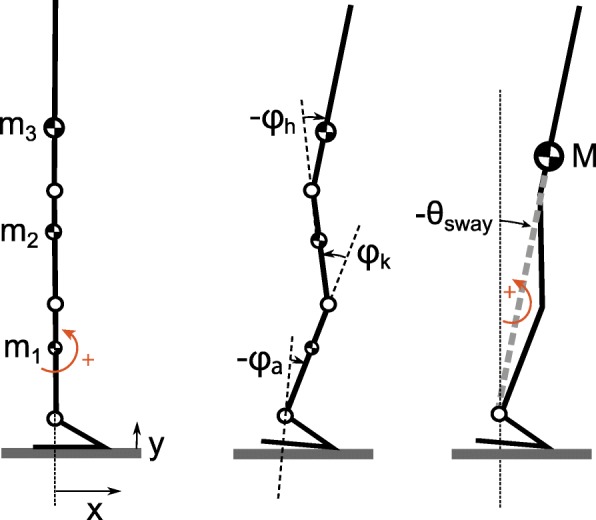
Computation of the body sway angle *θ*_*sway*_ based on a rigid link model. The variables *ϕ* are joint angles, *m*_*n*_ are segment masses and *M* is the total body mass located at the CoM. Left: model in baseline pose. The model is placed in a right-handed coordinate system. Segment rotations and moments are positive in counter-clockwise direction. Middle: the joint angles are defined as the angle of segment *n* minus the angle of segment *n*−1. Segment angles are zero in the baseline pose. Right: Based on segment angles and masses the whole body CoM can be computed. Similar to the segment angles, the body sway angle is positive in counter-clockwise direction

The body sway angle was estimated using a human model with four rigid segments: a foot, shank, thigh and head-arms-trunk segment (Fig. 3). The segments’ lengths, inertias, masses and CoM were estimated using the method described by Winter (2009) [31]. The orientation of the segments was obtained using the IMUs and expressed with respect to the baseline pose. For this baseline pose, subjects were requested to stand comfortably upright. Then all segment angles and the body sway angle were set to zero.

**Fig. 4:**
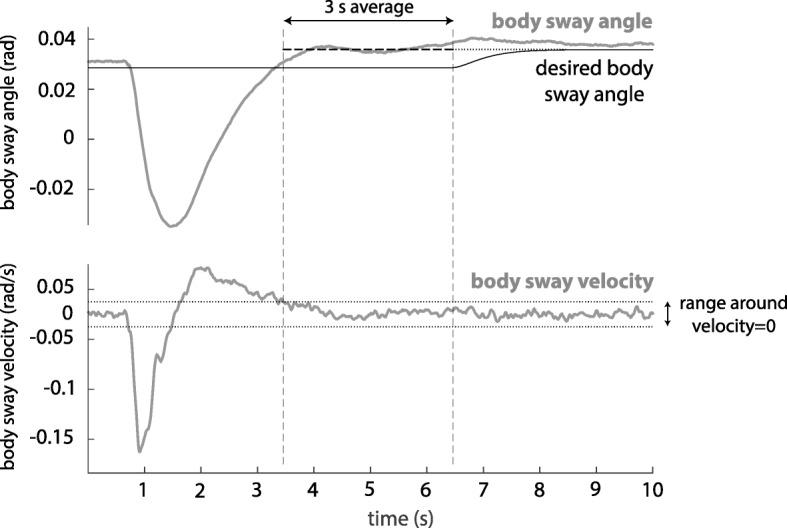
Adaptation of the desired body sway angle to a new steady pose. Top: body sway angle and desired body sway angle, bottom: scaled body sway velocity. The vertical dashed lines indicate the time frame in which the body sway velocity is approximately zero and the average body sway angle is computed. The desired body sway angle moves slowly to this average value

The desired body sway $\phantom {\dot {i}\!}\theta _{{sway}_{d}}$ was initially set to zero, matching the zero value of the body sway angle when a subject was in the baseline pose. During the experiments the desired body sway angle was adapted to match the body sway angle of the steady pose that subjects maintained between perturbations, allowing for natural variation in forward lean. This was done by updating the desired body sway when the subject’s body sway velocity remained close to zero for over 3 s. In a pilot test this duration had shown to be long enough to be sure that the subject had adopted a new steady pose. The updated desired body sway angle was then set to the average of the body sway angle during those 3 s. To prevent instantaneous changes in the desired body sway angle, the signal was passed through a second order low-pass filter with a cut-off frequency of 0.5 Hz Fig. 4 shows an example of how the desired body sway was adapted to a new steady pose.

To obtain the body sway velocity the body sway was differentiated and low-pass filtered using a second order Butterworth filter with a cut-off frequency of 10 Hz. The desired body sway velocity was set to zero.

The control gains were normalized to the subject mass *M* and CoM height *l*_*com*_:

$$\begin{array}{*{20}l} K_{P} &= {Mgl}_{com}\\ K_{D} &= 0.3\sqrt{{Ml}_{com}^{2}K_{P}} \end{array} $$

where *g* is the gravitational acceleration. The gain *K*_*P*_ was chosen such that the gravitational stiffness was compensated. As a result the subject was able to stabilize the system with a minimal amount of effort. The subject was assumed to provide the feed-forward torque to maintain the desired body sway angle himself. Gain *K*_*D*_ consisted of a normalization part $\sqrt {{Ml}_{com}^{2}K_{P}}$ multiplied by a factor 0.3. This multiplication factor was tuned empirically to maximize damping without causing unstable oscillations.

#### Virtual-ankle stiffness controller

The P _ankle_ is a proportional controller that takes the ankle angle *ϕ*_*A*_ as an input:

2 $$\begin{array}{@{}rcl@{}}  \tau_{ach} = K(\phi_{A_{d}}-\phi_{A}) \end{array} $$

where *τ*_*ach*_ is the output torque from the Achilles and *K* the proportional gain. The controller is similar to a simple ankle stiffness that tries to keep the ankle angle at its desired value. The desired ankle angle was initialized as zero, corresponding to the ankle angle in the baseline pose. Damping was provided by the inherent properties of the ankle of the subject wearing the Achilles.

The control gain *K* was chosen to be:

$$\begin{array}{*{20}l} K &= {Mgl}_{com} \end{array} $$

which is the same as the gain *K*_*P*_ of the body sway controller. Therefore, if subjects adjusted only their ankle joint angle, while keeping their foot flat on the floor, the stiffness provided by the virtual-ankle stiffness controller was equal to the stiffness provided by the body sway controller.

### Protocol

Each subject was tested under three control conditions: ZI, PD _com_ and P _ankle_ in a total of 10 trials. The order of the P _ankle_ controller (three trials) and the PD _com_ controller (three trials) was reversed after each subject. To prevent familiarization with the first controller influencing the second, a ZI trial was done in between. Furthermore, the PD _com_ and P _ankle_ controlled trials were preceded and succeeded by ZI trials to let subjects get used to the perturbation, collect baseline data and to check for fatiguing effects. The trial order for each subject is shown in Table 2.

**Table 2:** Experiment trial order. Subjects were coded S02 to S11

Subject Code	Controller Order
Sxx _*odd*_	ZI(0), ZI(1), P _ankle_(1), P _ankle_(2), P _ankle_(3), ZI(2), PD _com_(1), PD _com_(2),
	PD _com_(3), ZI(3)
Sxx _*even*_	ZI(0), ZI(1), PD _com_(1), PD _com_(2), PD _com_(3), ZI(2), P _ankle_(1),
	P _ankle_(2), P _ankle_(3), ZI(3)

Suffixes (0) to (3) refer to the trial number, where (0) indicates a practice trial

Before the first P _ankle_ and PD _com_ trial, subjects had one minute of practice time to feel the effect of the controller.

Before the experiments, subjects were instructed to try their best to maintain balance without stepping. They were instructed to cross their arms in front of their chests to prevent arm movement while they balanced. Furthermore, subjects were asked to stand still between perturbations with their weight evenly distributed on both feet and to return to their initial pose after a perturbation was applied.

During the experiments, the Pusher provided forward perturbations at the pelvis with two different magnitudes. The reference force was a rectangular pulse with a duration of 0.2 s and a magnitude of 0.08 *Mg* (N) (small perturbation) or 0.14 *Mg* (N) (large perturbation), where *M* is the mass of the subject and *g* the gravitational acceleration. Although the Pusher motors could not track the desired reference force perfectly, the provided pushes were consistent (Fig. 1b). In each trial subjects received 15 perturbations of each magnitude in a random order. The time between perturbations varied between 8 and 12 s.

After five trials subjects had a five minute break. Additionaly, subjects were given a rest when needed.

### Data collection and analysis

IMU data and joint encoder data were collected on the PC in the Achilles backpack at a sampling frequency of 100 Hz. Force plate, Pusher and EMG sensor data were collected on the XPC target PC at 1000 Hz. A signal composed of pseudo-random numbers and a time interval of 0.1 s between two samples was sent from the XPC target to the Achilles PC and logged on both systems. By computing the cross-correlation between the (resampled) signals, the delay between the systems was established and used for data synchronization.

The force plate and Pusher data were resampled to 100 Hz. To remove movement artifacts, the EMG data were first high-pass filtered in both forward and reverse direction using a third order Butterworth filter, resulting in a sixth order zero-lag filter with a cut-off frequency of 10 Hz. Then they were rectified by taking the absolute value, and low-pass filtered using a sixth order zero-lag filter with a cut-off frequency of 5 Hz to create an envelope.

To investigate the effect of the controllers on the standing balance of the subjects, the body sway, body sway velocity, ankle torques and muscle activity of the lower legs were analyzed. The body sway velocity was used to obtain a measure of the recovery time, that is, the subject was assumed to have recovered from a perturbation when the absolute body sway velocity was lower than 0.015 rad/s. This threshold was based on the computed variation in body sway velocity during quiet stance. The ankle torques can provide insight on how much the subject and device are contributing to maintaining balance. Therefore, the total ankle torque was derived using the center of pressure (CoP) in the anteroposterior (AP) direction. The CoP was computed from the measured moments and normal forces of the force plates. Since the location of the subject on the force plates was known, the CoP could be computed with respect to the time varying location of the ankle of the subject. For the estimation of the total dorsi/plantar flexion torque applied to the ankle, the mass of the foot was neglected. Then the total ankle torque

3 $$\begin{array}{@{}rcl@{}} \tau_{tot} = F_{Cy}p_{x}-F_{Cx}h_{A} \end{array} $$

where *F*_*Cy*_ is the vertical ground reaction force, *p*_*x*_ the CoP in the AP direction with respect to the ankle, *F*_*Cx*_ the horizontal ground reaction force and *h*_*A*_ the time varying ankle height. The torque applied by the Achilles was estimated from the leaf spring deflection, which was measured by differences between the motor and joint encoder readings. Subtracting this torque from the total torque resulted in the torque applied by the subject (the biological ankle torque). The total ankle torque, the torque applied by Achilles and the biological ankle torque were all normalized by a factor $\frac {1}{{Mgl}_{com}}$.

Data from the ZI, P _ankle_ and PD _com_ trials 1:3 were used; ZI(0) was excluded from the analysis to reduce the effect of subjects not being used to the perturbations. All data were split to extract the perturbation responses, and the first two perturbations of each trial were removed.

We computed the root-mean-square (RMS) of the body sway response from the start of a perturbation until the average time at which subjects had recovered. Therefore, the average recovery time was calculated over all subjects and all controllers. Additionally, we computed the RMS of the normalized total ankle torque, normalized biological ankle torque and the EMG envelopes, but then from the start of a perturbation until the average time at which the normalized total ankle torque had returned to a steady-state value. We assumed that the steady-state was reached when the absolute value of the derivative of the normalized total ankle torque was smaller than 0.02. This threshold was based on the variation in the derivative of the normalized total ankle torque during quiet stance. To compare the muscle activities of all the subjects, the means of the RMS values of the EMG envelopes in the P _ankle_ and PD _com_ trials were normalized by dividing them by the mean from the corresponding ZI trials.

#### Statistical procedures

Since not all data were normally distributed, we analyzed the data using non-parametric tests. Furthermore, both perturbation levels were analyzed separately. We used the Wilcoxon signed rank test to compare PD _com_ to the two other control cases by testing for differences in RMS body sway, recovery time, RMS torque and RMS EMG.

The overall significance level was set at *p*<0.05. To correct for two comparisons (PD _com_ compared to ZI and P _ankle_), for each test the significance level was set at *p*<0.05/2. To compute effect sizes, *z*-values were transferred to *r*-values, where *r*=0.1 corresponded to a small effect, *r*=0.3 a medium effect and *r*=0.5 a large effect [32]. All statistical procedures were carried out with SPSS Statistics 24 (IBM, New York, USA).

## Results

### Controller behavior

We investigated the general behavior of the virtual-ankle stiffness and body sway controllers by analyzing their inputs and outputs.

**Fig. 5:**
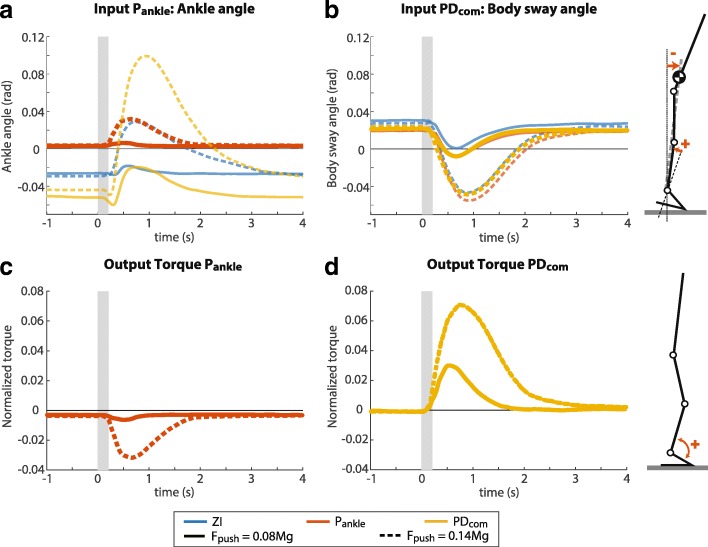
Controller inputs and outputs of a representative subject. **a** The ankle angle measured in the P _ankle_ trial, compared to the ankle angle measured in the ZI and PD _com_ trials. **b** The body sway angle measured in the PD _com_ trial, compared to the body sway angle measured in the ZI and P _ankle_ trials. **c** Normalized output torque of the P _ankle_ controller. **d** Normalized output torque of the PD _com_ controller. The grey boxes indicate the perturbation period. Similar results were obtained at a group level

Due to heel lift, the ankle showed plantar flexion in response to a perturbation, represented by an increasing ankle angle in Fig. 5a. However, the body’s CoM sways forward, which corresponds to the decreasing body sway angle in Fig. 5b. Therefore, the output torques of the P _ankle_ controller and PD _com_ controller are in opposite directions: a dorsiflexion torque for P _ankle_ (Fig. 5c) and a plantar-flexion torque for PD _com_ (Fig. 5d).

**Fig. 6:**
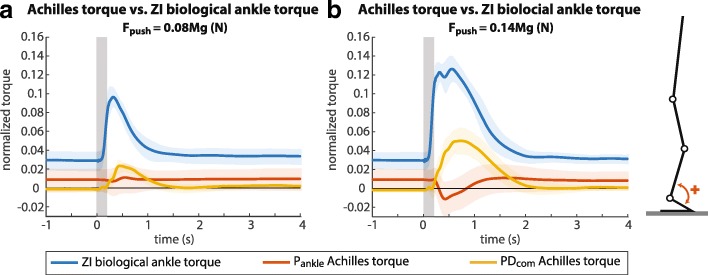
Normalized biological ankle torque in ZI compared to normalized torques provided by Achilles, mean over all subjects. **a** Torques in response to small perturbations. **b** Torques in response to large perturbations. The grey boxes indicate the perturbation period

A comparison of the normalized controller output torques and the normalized biological ankle torque in the ZI trial shows that for both perturbation sizes the torques generated by the PD _com_ follow a similar trend to the biological ankle torque in the ZI trial, while the torques generated by the P _ankle_ do not (Fig. 6). Despite these differences in support torques, subjects were able to maintain balance using each of the controllers.

### CoM kinematics

**Fig. 7:**
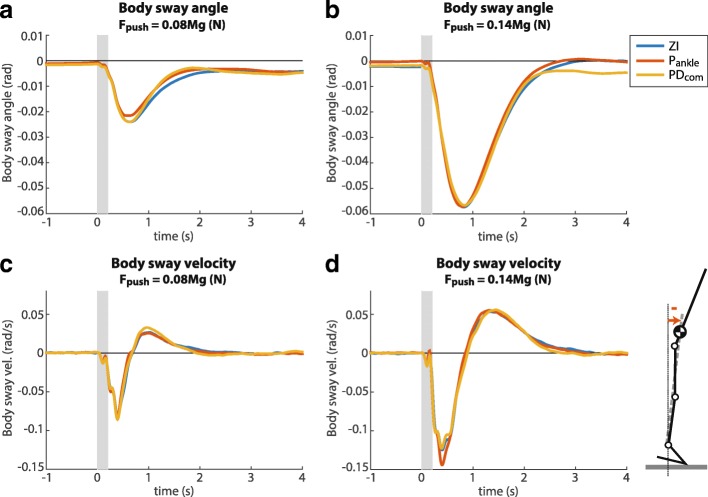
CoM kinematics, mean over all subjects. **a** Body sway angle in response to a small perturbation. **b** Body sway angle in response to a large perturbation. **c** Body sway velocity in response to a small perturbation. **d** Body sway velocity in response to a large perturbation. The grey boxes indicate the perturbation period

The amplitude of the body sway angle hardly varied with the controller used, for small and large perturbations (Fig. 7a, b). The results of the Wilcoxon signed rank tests showed no significant differences between the RMS body sway with the PD _com_ and the RMS body sway with the other two controllers (Table 3). The recovery time, derived from the body sway velocity, was also similar for all controllers for small perturbations and large perturbations (Fig. 7c, d, Table 3).

**Table 3:** Statistical test results from the Wilcoxon signed rank test for RMS body sway and recovery time

	Small perturbations		Large perturbations	
Variable	PD _com_ vs. ZI	PD _com_ vs. P _ankle_	PD _com_ vs. ZI	PD _com_ vs. P _ankle_
body sway	*z*=−0.255, *p*=0.846	*z*=−0.153, *p*=0.922	*z*=−0.357, *p*=0.770	*z*=−0.051, *p*=1.000
recovery time	*z*=−0.204, *p*=0.865	*z*=−0.510, *p*=0.643	*z*=−1.989, *p*=0.047	*z*=−0.306, *p*=0.785

### Ankle joint kinetics

**Fig. 8:**
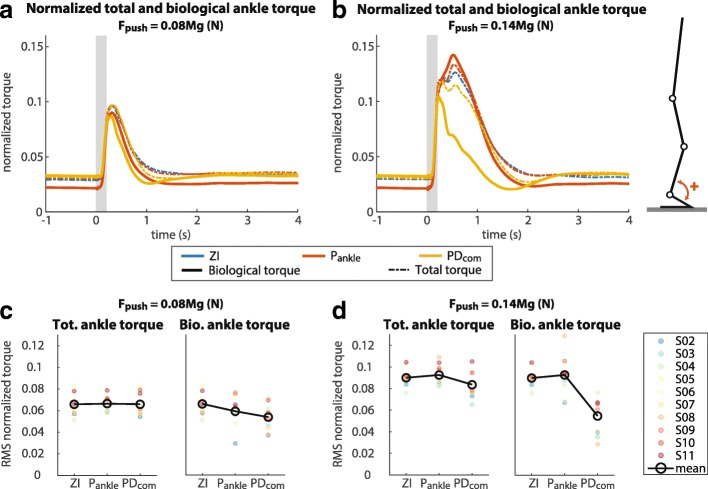
Normalized total ankle torque and normalized biological ankle torque. **a** Torques in response to a small perturbation, mean over all subjects. **b** Torques in response to a large perturbation, mean over all subjects. **c** Mean of the RMS of the normalized torques computed over the small perturbations for each subject individually. **d** Mean of the RMS of the normalized torques computed over the large perturbations for each subject individually. The grey boxes indicate the perturbation period

For both the small and the large perturbations, there was a decrease in normalized biological ankle torque for the PD _com_ compared to ZI and P _ankle_, while the normalized total ankle torque stayed approximately the same (Fig. 8, Table 4). The decrease in median RMS normalized biological ankle torque over all subjects was particularly large in response to large perturbations: a 29% decrease for the PD _com_ compared to ZI, and a 32% decrease compared to P _ankle_. The results of the Wilcoxon signed rank tests showed that the normalized biological ankle torque response to a large perturbation was significantly lower for the PD _com_ (*Mdn*=0.063) compared to ZI (*Mdn*=0.089), *z*=−2,701, *p*=0.004, *r*=0.60 and P _ankle_ (*Mdn*=0.093), *z*=−2,803, *p*=0.002, *r*=0.63. For the small perturbations the normalized biological ankle torque was only significantly lower for the PD _com_ compared to ZI, but not compared to P _ankle_ (Table 4).

**Table 4:** Statistical test results from the Wilcoxon signed rank test for RMS normalized total ankle torque and RMS normalized biological ankle torque

	Small perturbations		Large perturbations	
Variable	PD _com_ vs. ZI	PD _com_ vs. P _ankle_	PD _com_ vs. ZI	PD _com_ vs. P _ankle_
total torque	*z*=−0.866, *p*=0.432	*z*=−0.051, *p*=1.000	*z*=−1.886, *p*=0.064	*z*=−1.886, *p*=0.064
bio. torque	*z*=−2.497, *p*=0.010^a^	*z*=−0.866, *p*=0.432	*z*=−2.701, *p*=0.004^a^	*z*=−2.803, *p*=0.002^c^

^a^RMS PD _com_< RMS ZI

^c^RMS PD _com_< RMS P _ankle_

### EMG

**Fig. 9:**
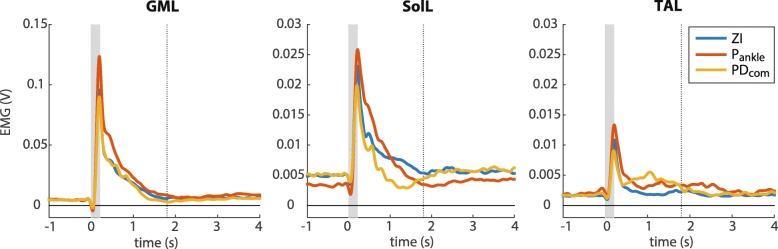
EMG envelopes of a representative subject, large perturbations. The grey boxes indicate the perturbation period, the RMS was computed from the start of the perturbation period until the dotted vertical line. Similar results were obtained at a group level

**Fig. 10:**
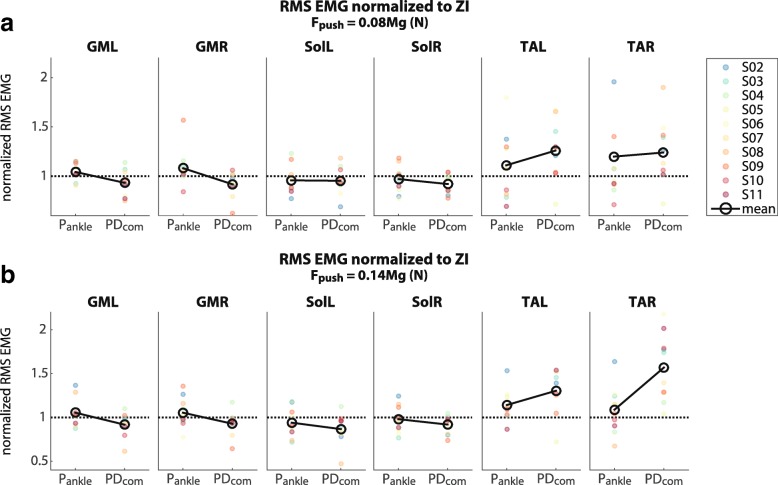
RMS EMG normalized to the RMS EMG for ZI condition. The dashed lines at 1 represent ZI. **a** Mean of the normalized RMS EMG over the small perturbations for each subject individually. **b** Mean of the normalized RMS EMG over the large perturbations for each subject individually

Generally, when the PD _com_ was applied, subjects showed a decrease in soleus activity and an increase in tibialis anterior activity with respect to ZI, as shown for a representative subject in Fig. 9. These results were also observed at a group level in response to large perturbations (Fig. 10b). In that case the median of the RMS soleus muscle activity of the left and right leg decreased on average with 8%, while the median of the RMS tibialis anterior muscle activity increased with 47% compared to zero impedance. The results of the Wilcoxon signed rank tests showed that the SolL activity significantly decreased using the PD _com_ (*Mdn*=0.88) compared to ZI, *z*=−2,293, *p*=0.02, *r*=0.50, while the TAR activity significantly increased (*Mdn*=1.56), *z*=−2,803, *p*=0.002, *r*=0.63. Test results for the opposite leg (SolR and TAL) were nearly significant (Table 5). In comparison with P _ankle_, PD _com_ also resulted in a consistent decrease in plantar flexor muscle activity and an increase in dorsiflexor muscle activity (Fig. 10), however this effect was only significant for TAR (Table 5).

**Table 5:** Statistical test results from the Wilcoxon signed rank test for normalized RMS EMG. RMS EMG was normalized to ZI

	Small perturbations		Large perturbations	
Muscle	PD _com_ vs. 1 (ZI)	PD _com_ vs. P _ankle_	PD _com_ vs. 1 (ZI)	PD _com_ vs. P _ankle_
GML	*z*=−1.362, *p*=0.203	*z*=−1.599, *p*=0.129	*z*=−1.599, *p*=0.129	*z*=−1.244, *p*=0.250
GMR	*z*=−1.988, *p*=0.049	*z*=−1.988, *p*=0.049	*z*=−1.988, *p*=0.049	*z*=−1.580, *p*=0.131
SolL	*z*=−0.968, *p*=0.375	*z*=−0.255, *p*=0.846	*z*=−2.293, *p*=0.02^a^	*z*=−1.274, *p*=0.232
SolR	*z*=−1.784, *p*=0.084	*z*=−0.633, *p*=0.557	*z*=−2.090, *p*=0.037	*z*=−0.561, *p*=0.625
TAL	*z*=−2.073, *p*=0.039	*z*=−0.889, *p*=0.426	*z*=−2.192, *p*=0.027	*z*=−1.362, *p*=0.203
TAR	*z*=−2.191, *p*=0.027	*z*=−0.561, *p*=0.625	*z*=−2.803, *p*=0.002^b^	*z*=−2.701, *p*=0.004^d^

^a^RMS PD _com_< RMS ZI

^b^RMS PD _com_> RMS ZI

^d^RMS PD _com_> RMS P _ankle_

## Discussion

The goal of this study was to develop a controller that provides balance support for an exoskeleton and its user. Therefore, we tested a body sway controller and a virtual-ankle stiffness controller on a pAFO, and assessed their effects on standing balance. The body sway controller showed a human-like torque profile—that is, a torque profile that has the same shape as the biological ankle torque profile with ZI—whereas the virtual-ankle stiffness did not (Fig. 6). Firstly, we hypothesized that when a human-like assistive torque was provided, the balance performance would improve. However, subjects did not use the provided support torques to respond to a perturbation quicker or to reduce the body sway. Secondly, we expected that the human contribution to the balance response would decrease. This was indeed the case, since subjects reduced their own contribution to ankle torque.

In the scarce literature on balance control of exoskeletons, no results have been reported on the effects on the biological ankle torque. Therefore, we compared our results with pAFO locomotion studies. For torque assisted walking, decreased biological ankle torques were reported [14, 15]. In these studies, the support torque profiles that were provided to the subjects had a similar shape as the biological torque profiles during unassisted walking. These findings are similar to our results on pAFO balance control.

In our work, the decrease in biological ankle torque was a combined effect of decreased soleus activity (8%) and increased tibialis anterior activity (47%). This is similar to the results of Collins et al. (2015) [14], who also reported decreased soleus activity and increased tibialis anterior activity (although in combination with affected gastrocnemicus activity), but different from the results of Kao et al. (2010), who only showed a decrease in soleus activity, without an increase of tibialis anterior activity [15]. For balance recovery from a forward perturbation the plantar flexor muscles are typically active. However, the increased tibialis anterior activity indicates co-contraction of the lower leg muscles. A possible reason for this co-contraction is that it could be an effective way to decrease the biological ankle torque, while limiting the decrease in joint stiffness. Since the EMG signal is not an absolute measure of muscle activity, it is not immediately clear whether the total muscle activity has increased or decreased. We expressed the RMS EMG with respect to ZI to be able to analyze the effects of the controllers on the muscle activities across subjects. This normalization impacts the interpretation of the 8% decrease in soleus activity and 47% increase in tibialis anterior activity, since the absolute changes in muscle activity are dependent on the muscle activity with ZI. Although in unassisted standing balance mainly plantar flexor muscles are active during recovery from a forward perturbation, Maki and Ostrovski (1993) reported a substantial amount of co-contraction for forward sway induced by backward platform perturbations, which increased with increasing perturbation magnitude [33]. Since the perturbations in our experiment were also large, we would expect substantial tibialis anterior activity with ZI. Therefore, it is not likely that the relatively large increase in tibialis anterior activity is a result of low muscle activity with ZI. Consequently, we cannot be conclusive about whether the total muscle activity increased or decreased through the concurrent changes in soleus and tibialis anterior activity.

Modifications in muscle activity can be feedback-driven or a result of feed-forward adaptation over time. In this study the reduction of the biological ankle torque when using the body sway controller could already be observed at the beginning of a trial and remained constant over that trial. This suggests that the changes in muscle activity are feedback-driven adjustments. Similar feedback responses were found for walking pattern adaptation in the presence of velocity-dependent resistance, but in combination with feed-forward adaptive strategies [34]. It is possible that feed-forward adaptation could also occur in standing balance with a controlled pAFO, but that the exposure to the controllers in this study was too short to demonstrate this effect.

Besides a decreased human contribution to ankle torque, we also found that the body sway in response to a perturbation was similar for all controllers (Fig. 7). It is not directly clear why the body sway stayed approximately the same, while the provided support torques were different. For example, in Functional Electrical Stimulation controlled standing, healthy subjects do change their body sway when different control strategies are applied [35]. There are several explanations for the unchanged body sway. Firstly, according to the inverted-pendulum model, the total torque must increase to reduce the body sway. In case of a large perturbation, it is not always possible to increase the total ankle torque considering that the CoP is already at the edge of the base of support. However, this does not hold for the small perturbations for which we saw the same effects. Secondly, subjects may prefer to reduce effort when they are confidently able to maintain balance. This preference of minimizing effort instead of body sway was also reported in standing balance identification studies, through comparisons between optimal and identified neural feedback gains [23, 36]. Lastly, subjects may need more training time to adapt their body sway response.

The body sway controller and virtual-ankle stiffness controller are not only different in the sense that they require different angles as an input. For one thing, for the sake of simplicity the virtual-ankle stiffness controller did not have a velocity dependent component. For another thing, for the body sway controller we allowed the desired body sway angle to adapt to a new steady pose, while for the virtual-ankle stiffness controller the desired ankle angle was kept constant. As a result of keeping the desired ankle angle constant, there was an offset from zero in the torque provided by the pAFO when the virtual-ankle stiffness controller was used (Fig. 6). Yet, we do not think that the virtual-ankle stiffness controller would perform better when a velocity dependent component was added to the control law, or when the desired ankle angle was updated to a new steady pose, since the support torques generated in response to a perturbation would still be small, or even in opposite direction (Fig. 6).

A practical implication of using a body sway controller is that the CoM location should be estimated on-line. Generally, exoskeletons are equipped with joint encoders. While this is sufficient for simpler controllers, such as the virtual-ankle stiffness, for a body sway estimation additional sensors are needed, which adds complexity to the setup. We used three IMUs and anthropometric data to make a CoM estimation, but simpler methods based on a single IMU have already been shown to give good results during walking [37].

Ultimately, our goal is to improve the standing balance of people with a spinal cord injury. Based on the results of the tests with healthy subjects, it is difficult to predict how paraplegics would respond to the provided support torques. Healthy subjects reduced their own ankle torques, but paraplegics, depending on the level of injury, typically cannot provide large ankle torques themselves. Therefore, a future direction is to test these balance controllers on an exoskeleton for people with a spinal cord injury who are not able to balance themselves.

## Conclusion

In this study we investigated the effects of a pAFO on the standing balance of healthy subjects. We found that when using a controller that uses body sway motion as an input, effective supportive torques could be provided. These support torques followed a similar trend to the ankle torques humans would provide in response to a perturbation. Although the CoM kinematics remained unaffected with the body sway controller, the biological ankle torque decreased when the body sway controller was implemented. This was a result of the combined effects of decreased soleus muscle activity and increased tibialis anterior muscle activity. Subjects were able to quickly adapt their ankle torques to the torques provided by the ankle exoskeleton. These results suggest a preference for reducing effort instead of further improving body sway.

## Abbreviations

AP: Anteroposterior
CoM: Center of mass
CoP: Center of pressure
GM: Gastrocnemius medialis
IMU: Inertial measurement unit
pAFO: powered Ankle-Foot Orthosis
RMS: Root-mean-square
Sol: Soleus
TA: Tibialis anterior
ZI: Zero impedance
